# Life in lockdown: a qualitative study exploring the experience of living through the initial COVID-19 lockdown in the UK and its impact on diet, physical activity and mental health

**DOI:** 10.1186/s12889-023-15441-0

**Published:** 2023-03-29

**Authors:** Tania Griffin, Elisabeth Grey, Jeffrey Lambert, Fiona Gillison, Nick Townsend, Emma Solomon-Moore

**Affiliations:** 1 Department for Health, University of Bath, Claverton Down, Bath, BA2 7AY United Kingdom; 2 Population Health Sciences, Bristol Medical School, Canynge Hall, 39 Whatley Road, Bristol, BS8 2PS United Kingdom; 3 School for Policy Studies, University of Bristol, 8 Priory Rd, Bristol, BS8 1TZ United Kingdom

**Keywords:** COVID-19, Lockdown, Physical activity, Diet, Mental health, Lifestyle behaviours, Qualitative

## Abstract

**Background:**

In response to the COVID-19 pandemic, the UK imposed a national lockdown prompting change to daily routines. Among behaviours impacted by the lockdown, diet and physical activity may be particularly important due to their association with mental health and physical health. The aim of this study was to explore people’s experiences of how lockdown impacted their physical activity, dietary behaviours and mental health, with a view to informing public health promotion.

**Methods:**

This phenomenological qualitative study used semi-structured telephone interviews. Interviews were audio-recorded and transcribed verbatim. Thematic analysis was conducted, guided by the Framework Approach.

**Results:**

Forty participants (28 female) completed an interview (mean duration: 36 min) between May and July 2020. The overarching themes identified were (i) Disruption (loss of routines, social interaction and cues to physical activity) and (ii) Adaptation (structuring the day, accessing the outdoor environment, finding new ways for social support). The disruption to daily routines altered people’s cues for physical activity and eating; some participants spoke of comfort eating and increased alcohol intake in the early days of lockdown, and how they consciously tried to change these when restrictions lasted longer than first anticipated. Others spoke of adapting to the restrictions using food preparation and meals to provide both routine and social time for families. Disruptions from the closure of workplaces resulted in flexible working times for some, allowing for physical activity to be built into the day. In later stages of restrictions, physical activity became an opportunity for social interaction and several participants reported intending to continue to replace sedentary means of socialising (e.g., meeting in cafes) with more active, outdoor activities (e.g., walking) once restrictions were lifted. Staying active and building activity into the day was seen as important to support physical and mental health during the challenging times of the pandemic.

**Conclusions:**

Whilst many participants found the UK lockdown challenging, adaptations to cope with the restrictions presented some positive changes related to physical activity and diet behaviours. Helping people sustain their new healthier activities since restrictions have lifted is a challenge but presents an opportunity for public health promotion.

**Supplementary Information:**

The online version contains supplementary material available at 10.1186/s12889-023-15441-0.

## Background

The COVID-19 pandemic resulted in unprecedented measures to restrict people’s movement and activity to control the spread of the virus. In March 2020 a ‘stay at home’ order was instated in the UK, referred to as ‘lockdown’. All non-essential services were closed and residents were permitted only to leave home for specified reasons such as the purchase of food and medical supplies, to exercise once-a-day, or to access emergency health care. The lockdown inevitably resulted in a change in working patterns, daily routines, physical activity and dietary habits [1]. Large scale studies with both healthy populations and disease-specific groups from around the globe have suggested a mostly negative impact of the pandemic on physical activity, shown by self-reported survey data [1, 2] as well as objective data such as step counts [1, 3]. Similarly, evidence from both cross-sectional and longitudinal survey studies worldwide highlight unhealthy dietary changes during the pandemic, with shifts towards increased snack and ultra-processed food consumption, alcohol consumption and decreased intake of fresh fruit and vegetables [4, 5].

The aim of this study was to explore how the impact of the UK lockdown on people’s diet and physical activity behaviours changed, and how this may have also influenced their health and wellbeing. Wellbeing is a broader construct than physical health alone, that is conceptualised as an ideal state of being or existence comprising two main elements: feeling good and functioning well [6] Mental health and wellbeing are known to have been negatively impacted by the pandemic. In terms of mental health; in the first week after the first UK lockdown, a survey of 36,520 adults in England found 47% and 52% reporting mild to severe symptoms of anxiety and depression, respectively [7]. Another UK survey study conducted one month into the lockdown found participants (N = 842) to have elevated levels of depression and anxiety symptoms, with those classified as ‘vulnerable’ or experiencing severe COVID-19 symptoms expressing particularly high levels of anxiety [8]. In terms of wellbeing, a survey study with Irish adults (N = 604) examined daily emotional wellbeing during the COVID-19 pandemic, finding that time exercising, going for walks, gardening, pursuing hobbies, and taking care of children were positively associated with wellbeing, while home-schooling children and obtaining information about COVID-19 were negatively associated with wellbeing [9].

Whilst studies have examined the impact of COVID-19 on diet, physical activity mental health and well-being, few have examined this in combination. Furthermore, much of the evidence related to physical activity, dietary habits, and mental health and wellbeing throughout the COVID-19 pandemic is based on survey evidence. Alongside this, qualitative research can offer context and personal experiences of the pandemic to help us better understand people’s behavioural responses [10]. For example, an interview study with adults aged over 70 in the UK highlighted the particular challenges they faced with regards to maintaining mental wellbeing and the coping behaviours they adopted in response [11]. Another interview study with primary care nurses provided insight into the selfcare strategies, including exercise, that this group implemented to help limit the effects of the increased stress and anxiety they felt as a result of the pandemic [12]. Much of the qualitative research so far has focused on specific groups, often with a heightened risk of COVID-19, or focused on single outcomes, such as mental health, to provide much needed insight into ways to support these defined populations. Alongside these studies, it is also important to consider the lived experience of people living in the community. We know from survey data the pandemic prompted behaviour changes, but to help predict long term maintenance we need to understand more about people’s reasons for making changes and their experiences of these new habits. These are insights that can’t be gained from survey data alone and can be helpful for setting future public health research and policy.

The present study used qualitative semi-structured interviews to explore the experiences of UK residents during the initial UK lockdown, with a specific focus on physical activity, diet, mental health and wellbeing. The insight gained from this study on people’s experience of the lockdown was intended to help inform future health promotion activities, both in current, less restricted times and if any future lockdowns are imposed.

## Methods

### Design

This phenomenological qualitative study [13] used semi-structured interviews Ethical approval was obtained from the University of Bath Research Ethics Approval Committee for Health (REACH) reference number: EP 19/20 041.

### Participants

Participants were a subgroup of respondents to an online cross-sectional survey study which is detailed elsewhere [14]. The survey was promoted through social media (Twitter and Facebook), a press release and interviews with local radio stations. The survey was open to anyone living in the UK during the national lockdown. At the end of the online survey, respondents were asked to leave their details if they were willing to take part in an interview. The list of volunteers was stratified by age, employment status and working conditions (e.g., working from home), and household set-ups (e.g., living with school-aged children). A maximum diversity sampling strategy [13] was employed to select participants from a range of lived lockdown experiences, for example, recognising that the lived experience of a retired participant would likely differ from that of working parents managing home schooling and home working, or parents of primary school aged children were likely to have different experiences from those whose children were in secondary school.

### Procedure

Participants who were selected for interview and provided an email address were sent the participant information sheet and consent form by email. The consent form was included for information and a note included to explain to the participant that if they chose to participate in an interview consent would be confirmed over the telephone. Participants were asked to read the attachment and if they were interested in taking part in an interview to respond to the email, or that a researcher would follow up with them in a few weeks. Thereafter, four researchers (EG, ESM, JL, TG), with experience of conducting research interviews, contacted potential participants to provide further information about the interviews and ask if the participant had any questions. If the participant agreed to take part, a convenient time for a telephone interview was arranged. Oral consent was taken at the start of the telephone call; each consent statement was read aloud to the participant who was asked to audibly confirm a response. A copy of the completed form was sent by email to the participant after the interview for future reference. Interviews were audio-recorded and transcribed verbatim; all identifying names and references were removed for anonymity. To provide context, the researchers conducting the interviews were in full time employment, of White ethnicity and experienced a variety of living circumstances during lockdown (living alone, with others and with young children).

### Interview schedule

The interviews were semi-structured and directed by a topic guide (see Additional File 1), allowing for iterative questioning and inviting participants to share their experiences of lockdown. Questions and prompts aimed to focus discussion on the impact of the lockdown on participants’ physical activity, diet, mental health and wellbeing, exploring any barriers or facilitators they experienced to being active, eating healthily and attaining positive mental wellbeing. To build rapport with the interviewees and encourage openness, the researchers took some time for an informal chat with participants before beginning the interview recording. Interviews were conducted between May and July 2020. In context, on the 26th of March, the first legally enforceable lockdown in the UK came into force which ordered people to “stay at home”. This was relaxed to a phased re-opening of schools in England on the 15th followed by non-essential shops reopening on the 23rd of June. On the 4th of July, more restrictions were eased, including the reopening of pubs, restaurants, and hairdressers [15].

### Analysis

Thematic analysis of the transcripts was conducted by two of the researchers (EG and TG), guided by the Framework Approach [16, 17]. First, 10% of the transcripts were reviewed independently. Second, the researchers met to discuss common themes they had identified and developed a coding framework. This framework was then applied to the remaining transcripts, using NVivo 12 (QSR International Pty Ltd. Version 12, 2018) software to help organise the data. The process was iterative with the researchers regularly discussing any new codes they identified that had not been captured in the original coding framework. When all transcripts had been coded, EG and TG reviewed and discussed the coding framework to ensure the themes were representative of the data. The final themes and their organisation were discussed among the whole research team.

## Results

Forty interviews were conducted, 70% of the participants (n = 28) were female and ages ranged from 25 to 75 + years. The majority were white ethnic group and lived in areas of low deprivation. Further details of the participant characteristics are provided in Table 1. Interview duration (following rapport building) ranged from 12 to 67 min (mean: 36 min). Throughout the results, interview quotes are presented followed by participant number, age group and gender (M or F), to demonstrate that the quotes presented represent a range of participants in terms of gender and age group. Data analysis was conducted with data from all 40 participants, and quotes selected from among the participants to show those most reflective of each subtheme. Two overarching themes were identified; (1) Disruption and (2) Adaptation, with subsequent sub themes (see Table 2).

**Table 1:** Characteristics of participants who participated in an interview (N = 40)

	n	%
**Gender**		
Male	12	30%
Female	28	70%
**Age category**		
25–35 years	7	18%
35–44 years	4	10%
45–54 years	10	25%
55–64 years	10	25%
65–74 years	5	13%
75 + years	4	10%
**Index of Multiple Deprivation decile**		
1–3 (more deprived)	3	8%
4–7	16	40%
8–10 (less deprived)	19	48%
Unknown	2	5%
**Ethnic group**		
White	39	98%
Other	1	2%
**Living with a long-term illness, health problem or disability?**		
Yes	2	5%
No	38	95%
**Living situation during first COVID-19 lockdown**		
Living alone	9	23%
Living with children aged 0–17	6	15%
Living with other adults but no children	25	63%
**Working situation during first COVID-19 lockdown**		
Not working	7	18%
Working from home	14	35%
Working outside home	6	15%
Retired	12	30%
Unknown	1	3%

**Table 2:** Themes and subthemes identified in the interviews

**Theme 1: Disruption to physical activity, diet and mental wellbeing**
(i) Loss of routine (ii) Loss of social interaction (iii) Cues for physical activity
**Theme 2: Adaptation related to physical activity, diet and mental wellbeing**
(i) Structuring the day (ii) The importance of the outdoor environment (iii) New forms of social support

### Theme 1: disruption to physical activity, diet and mental health and wellbeing

This theme captures the various ways in which participants reported that the lockdown restrictions impacted on their physical activity, diet and mental health and wellbeing.

#### Loss of routine

Several participants, most noticeably those whose working patterns were changed through lockdown, reported finding the loss of routine challenging. The loss of structure to one’s day, left some participants feeling they lacked a sense of purpose.

*“I do find that I’m getting up later. I find that I’m going to bed later as a result. And just generally I find that working, it’s a lot less structured than it would have been if I was in an office environment” [P22, 25–34 years, M]*

*“Having no structure at all which is what I have at the moment… it makes you a bit uncertain, when you’re used to having rules and structures and have none at all, it’s quite difficult… Being furloughed and not having any work to do means that I have no structure at all to my day. The only structure in my day is that I wake up at some point and I have to eat and at some point I have to go to sleep but there isn’t anything in between”. [P16, 45–54 years, M]*

#### The loss of social interaction

Lockdown restrictions prohibited mixing with others outside one’s household for social purposes. This loss of social interaction was reported by many participants to be challenging, and noted by some to negatively impact their mental health. Among participants who lived with others, some mentioned placing more emphasis on planning and preparing meals and eating together, seeing this to be an important time for social interaction (see Theme 2). Missing socialising and eating out was frequently mentioned; those who lived alone particularly felt this loss and reported feeling lonely or isolated, which made abiding by the lockdown rules hard.

*“I have felt lonely and I think touch is hugely significant and the fact that when I see my friends or relatives I can’t give them a hug you know, particularly because I live on my own, it’s quite, it’s really depressing”. [P15, 65–74 years, F]*

*“the lockdown rules because they’re not, they just don’t take into account the impact on people who live on their own… the only people I know who don’t break the rules are people who are in families”. [P16, 45–54 years, M]*

#### Cues for eating and physical activity

In the early days of lockdown, all participants expressed that they had experienced disruption to their environment and in turn, many spoke of how cues for their daily habits for both eating and physical activity were both positively and negatively impacted. Firstly, in relation to diet, the change in working routines and locations in some cases removed cues for incidental eating opportunities, such as not being able to drop into fast food restaurants when journeying to and from work or less exposure to commercial eating outlets in the working environment.

*“…[pre COVID-19] I did tend to grab, like, a Greggs vegan sausage roll or something like that just because I was walking past it, and obviously everybody got coffees, and if you get coffee sometimes you get a cookie to go with it ... [in lockdown] I just had my healthy breakfast in the house and I didn’t grab stuff because I was peckish. [P05, 35–44 years, F]*

In contrast, others found additional cues for eating in their home environment, such as a closer proximity to the kitchen while working, leading to an increase in snacking.

*“ when you’re working from home I think there is an element of boredom, especially where I was sat working where I was staring at the fridge … even if you’re making yourself a tea or coffee and you see something in the fridge and you start eating it, … if you are out on the go, you know, foods not always as important, I became a lot more conscious about controlling urges” [P13, 45–54 years, M]*

There were also positive and negative changes to cues for physical activity during the lockdown. The change in working patterns for some participants offered a more flexible approach to the working day, presenting additional opportunities to be physically active, be it during an extended lunch hour or in time that would otherwise be spent commuting.

*“It means I can cycle in my lunch hour, so if I don’t get out after work I can at least be getting out during the week in my lunch hour”. [P23, 45–54 years, F]*

The government announcement that exercise was a permitted reason to leave the house seemed to encourage people to capitalise on this opportunity.

*“When it was announced you could go out for an hour’s walk, we did that come what may, even on the days at the beginning when it was raining. Whereas prior to lockdown, if it had been raining, we’d never have dreamt of going out for a walk. But because it was almost as though everything had been taken away from you, but actually they had given something back in the form of an hour’s walk”. [P02, 65–74 years, F]*

Conversely, a few participants mentioned a decrease in activity when mostly based at home, or a reduction in incidental activity, such as walking to or around the workplace, and the added pressure of childcare and/or home schooling led to a reduction in time and/or motivation for exercise.

*“my husband used to walk to work and he used to do between 10,000 and 15,000 steps a day. And now that he doesn’t go into the office he’s doing no exercise at all…he’s not motivated in terms of looking after himself or anything. So it’s had an absolutely disastrous impact on his exercise” [P17, 55–64 years, F]*

### Theme 2: adaptation related to physical activity, diet and mental wellbeing

This theme covers the ways in which participants adapted to their new circumstances, some of which evolved through the lockdown as it became evident that restrictions were to last longer than initially thought.

#### Structuring the day

In acknowledgement of their loss of routine, some participants explained how they adapted by using their one permitted outing of the day to try to ensure their days had some structure to break up the monotony; for many this was also an important coping strategy, to help their mood and overall wellbeing.

*“…we will all go out … to the beach or the park or somewhere with not many people to go and wander around in. That was quite exciting in a way when we got used to that, we’d sort of look forward to 3 o’clock... there was a lot more of nothing, nothing, nothing, nothing, something, nothing, nothing, but it felt like, it made it feel a bit special then” [P05, 35–44 years, F]*

While there was less of a change in routine amongst retired participants, their social activities were often reduced. This led many to increase their solitary hobbies and place greater importance on the remaining excursions that were allowed within the restrictions.

*“I am retired but I am one of the fit ones in the village. So there is a couple close to me, I go out and get a prescription for them and when I go shopping, help them with bits and pieces… No, I mean I’m fortunate my son rings and ‘Dad do you want anything’ and I say ‘No’. Goodness, that’s my one highlight of the week going outdoors, to go shopping”. [P09, 65–74 years, M]*

*“In the month of May I walked five marathons and I climbed 15,000 feet. … well, I had nothing else to do … I try and do something in the morning and break the day up a with decent long walk in the afternoon and then settle down in the evening. I think I’ve probably watched more rubbish television than I have in the rest of my life.” [P14, 75 + years, M]*

Physical activity was often mentioned as an important means of providing structure to participants’ days, where their normal routines had been disrupted. Physical activity provided an opportunity to take time for oneself, especially for those in busy households, to see others, either socially or in passing, or to have a change of scene.

*I guess making sure we go for a walk at lunchtime and actually talk to other members of the family rather than just existing in the same house, I think that’s really helped because obviously I’m like I am checking that everyone’s okay and you know they’re checking I’m okay as well so it’s quite nice to do that and I think having the little games in the garden it’s just a bit of fun at the end of a boring day.[P01, 45–54 years, F]*

Some participants tried engaging with online exercise classes during the lockdown, either to replace their usual gym sessions while unable to access gyms or to overcome the decrease in incidental activity. For some, this was a positive experience that they hoped to continue beyond the lockdown.

*“one of my friends, he’s a yoga instructor and he pointed out that [app] was a free, they were running it for free... so I downloaded that and started using it ...I think I’m going to try and keep going with the yoga actually once lockdown ends... I have found that it’s quite a good way of sort of clearing your head [P03, 35–44 years, F]*

However, others struggled to engage with exercise in the home setting and felt the loss of social interaction that they gained in a gym.

*“I enjoy my PT, because I go [to the gym], I throw around heavy weights and I come out of there feeling powerful... it was a really good place the gym I go to, and I’ve really missed it”. [P20, 25–34 years, M]*

*“I’ve done a lot of online classes from home but yeah it’s not the same, really as going, cos it’s sort of in your living room or whatever trying to fit in around everybody else in the house, and it’s, I don’t associate exercise with being at home particularly so it’s much harder to kind of push yourself” [P18, 45–54 years, F]*

Food was mentioned frequently in relation to routine, and for some it was used as a coping strategy, providing a source of comfort or distraction or structure to the day. Participants discussed mealtimes providing clear boundaries between work and leisure time, and an opportunity for social interactions within the household. Taking time to prepare food trying new recipes or baking, and eating together was a source of enjoyment.

*“I kind of welcomed the novelty of life having to be different and simpler and like having weird dinners… there is always stuff in the back of your cupboard that you would like ignore for like a year... I was quite up for it” [P05, 35–44 years, F]*

*“we made Sushi yesterday for the first time, it was really good fun, and having the time to do that sort of thing is lovely” [P12, 45–54 years, F]*

*“I’ve been cooking a lot more, because can’t go out to eat … food has become more important during lockdown…you haven’t got other sensory stuff going on, your only sensory thing probably is food” [P06, 35–44 years, F]*

For some, the disruption to their routines prompted the adoption of dietary practices they normally reserved for holidays, such as increased snacking, eating larger and more elaborate meals, and increased alcohol consumption.

*“the meals have become a much bigger part of our life…probably be a bit more elaborate in what we make and bit more of a holiday diet rather than a day to day diet you know, um, when we go away on holiday we tend to eat a lot more and I’ve found that we’ve been doing that through lockdown”. [P10, 45–54 years, F]*

*“I started putting sugar on my porridge again, umm sugar in my coffee, lots of cakes and biscuits through the day…I was buying lots of treaty food … I was buying far more than we would actually normally eat, but I felt like I needed to nurture or nourish something. There was just that need to comfort myself I think” [P21, 25–34 years, F]*

However, despite this initial disruption, once evident lockdown was going to last longer than a few weeks, many people reported re-adjusting their diets, especially with reference to alcohol intake.

*“since lockdown … we were drinking more, but again that’s on the downturn again as well. My husband has given up all together and I am limiting myself to one drink at five o clock... all of the things that got out of hand are now settling down more” [P07, 55–64 years, F]*

As well as physical activity and meals, simply making time for oneself was acknowledged as an important factor to include in the day. During a time of anxiety, such as the early days of the pandemic, having time for oneself was felt by some to be particularly important for mental wellbeing.

*“I think if lockdown’s taught me anything, it’s taught me the benefit of having ‘me time’… It’s a global pandemic, there’s things going on that I can’t have any influence over. So for me it’s sort of taught me to realise when I’m perhaps not coping so well and take that time to just either sit and read a book or have a bath or sit and watch some sort of trash TV programme. And just not sit and think about everything that’s going on. I don’t think that’s something I necessarily appreciated before lockdown”. [P22, 25–34 years, M]*

#### The importance of the outdoor environment

The outdoor environment played an important role for many participants, having a positive impact on mood and wellbeing. Enjoyment in walking was discussed, as was exploring the local area and observing nature. Many spoke of engaging with, or taking up new, hobbies outdoors such as birdwatching or gardening.

*“we’ve found some new places within walking distance from our house that like we’ve never really noticed or never been to before, so it’s been quite nice in that that respect”. [P01, 45–54 years, F]*

*“I am sure a lot of people are the same where they’ve done a lot of DIY... and I’ve taken up building stuff out of wood, and gardening and growing vegetables, and all sort of things that we’d never done before, so that’s been really positive”. [P10, 45–54 years, F]*

It is important to note that during the first lockdown the UK experienced a long period of fine weather, ideal for enjoying outdoor activities. Participants acknowledged this had a positive impact on their mood and encouraged them to be active outdoors, conceding that on wet or colder days, their motivation for outdoor activity decreased.

*“The weather helped, cos it was so lovely... we do like walking but I think if the weather had been like it has the last day or two obviously that would’ve caused a few more issues of getting out. [P11, 45–54 years, F]*

Having an opportunity to leave the house and access outdoor space was also seen as important for providing a change of scene from the home environment.

*“when I was able to go out …only to walk to do some shopping, that was a real joy just to get out and walk in the fresh air rather than being stuck inside”. [P04, 55–64 years, M]*

*“I’ll go for that walk, part of it is making sure I’m keeping active but also it’s that just taking some time out to not think about everything that’s going on in the world at the moment. And I think it’s important for my mental health to sort of get that fresh air and be by the water… my front room is now my office as well so it’s that separation of work and life space has sort of become very, very blurred” [P22, 25–34 years, M]*

#### New forms of social support

The importance of community, supporting friends and neighbours locally, was discussed amongst participants, such as collecting shopping or dropping off baked goods. When meeting others from different households outside was permitted, being physically active together, such as going for walks or cycle rides, was seen as an important opportunity for socialising, taking the place of more sedentary meetings at cafes, pubs etc.; activities which were not allowed at the point of lockdown, but which may have been the default prior to lockdown. Participants spoke of hope for this to continue but acknowledged likely barriers.

*“I went for a walk this morning with a friend... that’s kind of not been part of our social lives before, you know, it’s sort of seeing each other in the evening to go for a drink, or to go see a play or whatever, but now I would like to be able to carry on saying to people oh can we go for a walk? Obviously going into autumn and winter that might be- but I think hopefully it will become engrained enough of a habit that it’s something I want to carry on doing. [P12, 45–54 years, F]*

*“The walking with my friends now that is really nice, we’ve really loved that and I really hope that that stays….it was something that I was doing before but not as frequently, so there’s kind of 2 groups of friends and my sister, so I’ve been trying to do walks with, you know 3 nice big walks with those people each week. I don’t think it’s sustainable as everyone gets busier but I’d really like for those long walks to carry on in some shape”. [P19, 35–44 years, F]*

## Discussion

This study provides insight into the impact of the UK lockdown on physical activity and dietary behaviours. The inevitable change in daily routine due to the lockdown resulted in adjustment to both dietary and physical activity behaviours which for some participants was associated with changes (both positive and negative) in their perceived mental health and wellbeing.

Our findings show the short-term changes participants made to their diet and physical activity at the start of lockdown, as well as how conscious decisions were made to adapt their behaviour in more sustainable ways as it became clear that the lockdown period would last longer than anticipated. Past research has detailed how behaviour is more within intentional control when environmental cues that drive habits are disrupted [18–20], and these findings provide a case study of the types of considerations people make in forming and enacting intentions towards new habits the extreme case of disruption that the COVID-19 pandemic brought about. As lockdown progressed, participants displayed a need to establish new daily routines to support their wellbeing, and diet and physical activity were key ways of providing this routine. This supports pre-pandemic research on the benefit of routine and habits in making healthy choices [21, 22]. The adoption of less healthy behaviours appeared to be driven in our sample by emotions such as anxiety, boredom, a desire for comfort, as well as more instrumental barriers such as the lack of access to facilities and social support. Adopting healthier behaviours seemed to be associated with participants finding enjoyment in new activities, being motivated to take action for personal wellbeing, and having the time and resources to adopt healthier practices.

In some cases, participants expressed the desire to retain their new, healthy and enjoyable habits beyond the end of pandemic restrictions. However, it is likely this will be challenging for many due to a change in both opportunities (such as having time at home, and fewer conflicting responsibilities), and the re-introduction of the environmental cues that promoted former habits (e.g., driving rather than walking, eating out). For example, for some, a return to the workplace may have prompted old, less healthy habits, potentially over-riding any healthy intentions formed during lockdown. However, there may still be an opportunity to capitalise on some of the more positive experiences some people had during the pandemic related to diet and physical activity, whereby they may be more open to change than they would have been previously, giving interventions to promote physical activity and dietary change at a population level a greater chance of success [23]. From a public health perspective, environmental interventions and policy action to increase the availability and accessibility of healthy foods (and decrease the prevalence of unhealthy food) and encourage active commuting could help to provide cues for some people towards healthier habits [24]. Flexible working practices have also been shown to support positive health and wellbeing [25, 26] and may well support the ways in which people have found ways to incorporate local exercise into their day, however, it is recognised that flexible working is not always practical or possible and its suitability will vary by both employer and employee circumstance. Similarly, future work is needed to identify opportunities for physical activity to be built into the working day, especially for professions where flexible working is not possible. This has been explored by Ryde et al. (2020) [27] who identified that, whilst the likely benefits were acknowledged by employee and employer, there were significant barriers, such as workload and culture.

Food provided a focal point of the day for many, and flexible routines allowed more time in planning, preparing and eating meals within the household, giving structure to the day and, for some, social interaction. It seems plausible to suggest this could have led to acquisition of skills in food preparation which can hopefully be sustained. Similar to findings in the literature [4, 5], an initial change toward less healthy eating behaviours was reported, however this seemed to be temporary and consciously addressed, moving back towards previous habits as lockdown progressed. Previous research on the association between family meals and health outcomes [28, 29] lends support for the positive impact of eating together within multiple occupancy households observed in this research. Since the COVID-19 pandemic prompted enforced changes, and rapid adaptation to changed working patterns, it may present an opportunity to reflect on these changes, with emphasis on the importance of positive lifestyle behaviours for health, wellbeing and disease prevention. As the present study sample was predominantly affluent, with 30% of retired individuals, it is worth noting that for less affluent groups and those working irregular hours, eating together as a household and negotiating changes to working patterns may be less easy.

The increased time spent in the outdoor environment and appreciation for nature reported by participants is similar to that seen in New Zealand [30] and corroborates data reported by the UK Office for National Statistics (ONS) that showed a rise in use of parks and green spaces in 2020 compared to previous years [31]. While the ONS report also included survey findings suggesting that people placed an increased importance on green and natural spaces for their wellbeing following the onset of the COVID-19 pandemic, it also highlighted the inequality in access to such spaces. Having limited access to natural spaces of sufficient quality is likely to negatively impact people’s wellbeing in multiple ways; it limits their ability and motivation to exercise outdoors and prevents them benefitting from the stress-relieving effects of nature [32]. The increased appreciation for the outdoors brought about by the COVID-19 pandemic adds support for local authorities or charitable organisations, to drive improvements to access of green spaces.

The important interplay between mental wellbeing and lifestyle behaviours was also spoken of by participants: feeling anxious and depressed was reported to be linked to lack of motivation for exercise and turning to food for comfort. The restrictions on social interaction were challenging for many participants but particularly for those living alone and there was some indication that the lockdown caused some participants to reconsider their values and priorities, placing greater emphasis on (or simply acknowledging the importance of) social connection. Making use of the permitted exercise outing presented an opportunity for social interaction, whether this was to say hello to strangers in passing or, when lockdown rules eased, meeting up with friends and family. These findings suggest that encouraging and facilitating active social gatherings could be a useful area of future research, identifying whether it is a practical and effective approach to promoting both mental and physical health and wellbeing across a range of diverse settings. The UK government has been tracking population mental health and wellbeing throughout the pandemic, revealing a continually fluctuating nature [33], which is likely in turn to impact people’s health behaviours. Our findings, therefore, lend support to calls for further investment in public mental health support [34], policies to support emotional wellbeing, and emphasises an opportunity to promote physical activity, a known treatment for poor mental health and wellbeing [35].

Participants in this study discussed a heighted awareness through lockdown of the contribution that food and physical activity can have towards their daily experience, and how these were considered important to support their physical and mental health and wellbeing. For example, people learned new routes and opportunities within their local area, tried out new forms of socialising (e.g., walking, cycling with friends) that were cheap and enjoyable alternatives to previous habits (e.g., eating and drinking out), gained confidence in new skills and activities, and found ways of being together enjoyably as a family (e.g., cooking together). These findings parallel those found in a qualitative study of older adults in Mauritius whereby a positive of the lockdown experience was strengthening family bonds, taking part in activities together and enjoying time for hobbies [36]. Self-efficacy, motivation and health status have been associated with physical activity in past research [37]. The accumulation of experiences that have improved knowledge, skills and confidence provides opportunity for targeting interventions that reinforce, and remind people to support the continuation of some, or all of these activities longer term, to promote and support healthy lifestyle habits.

### Strengths and limitations

A key strength of this study is that it provides a qualitative insight into lifestyle experiences across a range of participants residing in the UK through the lockdown measures. A number of limitations are acknowledged. First, the timing of interviews meant that COVID-19 lockdown restrictions evolved through the data collection period (May - July 2020). Participants who were interviewed as restrictions were easing were asked to reflect back on their experiences in the earlier lockdown period, as well as on the present day, however, there may be risk of recall bias amongst participants interviewed later in the study. It should be noted that during the first lockdown the weather was warm and pleasant; experiences of later lockdowns during winter months, in particular on our key areas of interest (physical activity, diet and mental health), may have been different. Few of our participants resided in areas of high sociodemographic deprivation and most were female and of White ethnicity, which we recognise limits the generalisability of the findings. While we did not gain individual-level income data, it is likely that our sample is not representative of underserved and minority ethnic groups in the UK amongst which the impacts of the COVID-19 pandemic are known to be worse [38, 39].

## Conclusion

This study provides insight into the reasons behind people’s fluctuating health behaviours over the period of nationally imposed restrictions. The findings highlight the impact, both positive and negative, of restructured days and negotiating changes to working patterns. They show the multiple roles of food and food preparation in coping and adjusting to restrictions, and how people’s understanding of the importance of physical activity and diet for maintaining their wellbeing developed and shaped their behaviour over time. This insight could have relevance to public health attempts to promote or support people to maintain positive behaviour changes, such as replacing sedentary forms of socialising for physical activities which incorporate a social element, building on the experiences identified over the period of national restrictions and lockdowns.

## Electronic supplementary material

Below is the link to the electronic supplementary material.

Supplementary Material 1

## Data Availability

The datasets used and analysed during the current study are available from the corresponding author on reasonable request.
