# Supplementary material for: Life in lockdown: a qualitative study exploring the experience of living through the initial COVID-19 lockdown in the UK and its impact on diet, physical activity and mental health
Source: BMC Public Health. 2023 Mar 29;23:588. doi: 10.1186/s12889-023-15441-0 (PMC10052307; doi:10.1186/s12889-023-15441-0)
Supplement: Supplementary file 1 — Supplementary Material 1 [file 12889_2023_15441_MOESM1_ESM.docx]

**Additional File 1:**

Topic guide for semi-structured qualitative interviews.

General

- Please can you tell me about your general day to day routine during the lockdown period and how does this compare to your normal routine?
- Can you tell me how your health has been during the lockdown period and how does this compare to normal?

Diet and nutrition

- Thinking about you individually - can you tell me about your normal dietary habits before lockdown?
- Has your diet changed at all during the lockdown period?
- How have you been accessing food through the lockdown period?
- Have you found it difficult to get the food and drink items you would normally buy from the shops during the lockdown period?
- Do you think your diet will change in any way once the lockdown is lifted?

Physical activity and sedentary time

- Can you tell me about your normal activity and exercise routine before lockdown?
- Prior to lockdown, how many hours a day do you think you spent sitting down or standing still?
- Has your activity and exercise routine changed during the lockdown period?
- Have you felt any differently about physical activity, and its importance to you, over the lockdown period?
- There has been an upsurge in the number of exercise routines available online. Are you aware of these?
- Do you think your daily activity routine will change in any way once the lockdown is lifted?

Mental health and wellbeing

And finally, can you tell me how your mood [or mental wellbeing] has been over the lockdown period compared with normal?
